# *In Situ* Monitoring of Microwave Processing of Materials at High Temperatures through Dielectric Properties Measurement

**DOI:** 10.3390/ma9050349

**Published:** 2016-05-07

**Authors:** Beatriz Garcia-Baños, Jose M. Catalá-Civera, Felipe L. Peñaranda-Foix, Pedro Plaza-González, Gabriel Llorens-Vallés

**Affiliations:** ITACA Institute, Universitat Politècnica de València, Camino de Vera s/n, Valencia 46022, Spain; jmcatala@dcom.upv.es (J.M.C.-C.); fpenaran@dcom.upv.es (F.L.P.-F.); pedplago@upvnet.upv.es (P.P.-G.); gabllova@itaca.upv.es (G.L.-V.)

**Keywords:** microwave, high temperature, process monitoring, dielectric properties, heating, ceramic materials

## Abstract

Microwave-assisted processes have recognized advantages over more conventional heating techniques. However, the effects on the materials’ microstructure are still a matter of study, due to the complexity of the interaction between microwaves and matter, especially at high temperatures. Recently developed advanced microwave instrumentation allows the study of high temperature microwave heating processes in a way that was not possible before. In this paper, different materials and thermal processes induced by microwaves have been studied through the *in situ* characterization of their dielectric properties with temperature. This knowledge is crucial in several aspects: to analyze the effects of the microwave field on the reaction pathways; to design and optimize microwave-assisted processes, and to predict the behavior of materials leading to repeatable and reliable heating processes, *etc*.

## 1. Introduction

Microwave processing of materials is a well-known and established technology. In the last decade, microwave energy applications have extended towards processing at increasing temperatures (>1000 °C) [1]. Promising results have been obtained in the laboratory, e.g., materials with innovative properties, new reaction routes, ultrafast heating, efficient processing, *etc*.

In this context, there is a necessity to increase the transfer of these benefits to an industrial scale. However, several barriers hinder the scalability of the results obtained at laboratory scale. In particular, the need arises to include new elements such as materials for the transport system or thermal insulation in the microwave system. The microwave energy absorption by such elements and its variation with temperature may deteriorate the performance of the microwave applicator. Nevertheless, there is a generalized shortage of available data regarding the dielectric properties of these materials at high temperatures [2].

Another barrier is the lack of understanding of the heating mechanisms when materials are subjected to high frequency fields. The effects on the materials’ microstructure are still unknown, and very often it is not possible to predict the materials’ behavior during the heating process [1,3]. This problem is accentuated when mixtures of materials are processed, or when materials experience changes and transformations at certain temperatures, because their interaction with microwave fields is even more complex. Again, precise knowledge about the materials’ dielectric properties (measured under microwave fields), and their variations with temperature is essential to perform an adequate design of the heating process.

There are numerous studies on the measurement of dielectric properties of materials and their evolution with temperature. As an example, a comprehensive study was reported in [4] by Von Hippel and contributors. Table 1 shows a brief summary of the work done on this subject, with the corresponding references.

In one possible configuration, the dielectric measurements are conducted in the frequency range of KHz–MHz. Measurement of dielectric properties is obtained from impedance measurements, leading to simpler measurement devices (data collections can be found in [5,6]). The main disadvantage is that these studies do not include measurements in the standard ISM frequencies (915 MHz, 2.45 GHz). In general, the values of dielectric properties cannot be extrapolated to that frequency range because the physical mechanisms contributing to the material dielectric response (rotation of dipoles, movement of charges, *etc.*) are different depending on the frequency.

Regarding the maximum temperature, results of dielectric properties are often provided up to temperatures of only several hundred degrees. This temperature limitation is mainly due to increasing radiation losses at higher temperatures and also due to practical limitations of commercially available measurement devices.

To heat the sample, one method consists of using an external heater, and once the sample reaches the desired temperature, it is inserted in a measurement cell to determine its dielectric properties. This technique presents several disadvantages. On one hand, the sample cools down during its way from the heater to the measurement cell. Some authors try to model this cooling with complex thermal models but it still represents an important source of uncertainty in the results. However, the main problem appears when the materials suffer a rapid or irreversible change at certain temperature. When the sample cools down, the relationship between the change of dielectric properties and the corresponding temperature can be lost.

To avoid these problems, another method consists of maintaining the sample in the measurement cell, and the cell is heated together with the sample. This configuration has limited applicability regarding the maximum heating rate and the measurable range of dielectric properties.

A third method is heating the sample to the maximum desired temperature, and measuring its dielectric properties during the cooling cycle. This technique has the evident drawback that dielectric properties generally differ in the heating and cooling cycles, especially if changes or transformations occur in the material.

Taking into consideration the problems related to the conventional heating of the samples, a different methodology relies on the use of microwave fields both to heat and to characterize the dielectric properties. In this case, two configurations are possible. In the first configuration, one field mode is used to heat and measure the sample permittivity. In the second configuration, two independent modes are used, one to heat the sample and the other one for the dielectric measurements. In both options, the response of the measurement cell (in particular, the resonance frequency and the quality factor of the heating mode) are strongly dependent on the sample dielectric properties, which are changing with the increasing temperature. Thus, a tracking mechanism is needed to adjust the response of the measurement cell and adapt it to the changes in the sample properties. This tuning process is often made by mechanical components, which severely limit the temperature and dielectric properties measurable ranges in these systems.

To overcome these drawbacks, [29] reports a new dual-mode technique which allows the dielectric characterization of the sample under microwave heating, without the need of mechanical adjustments of the measurement cell during the heating process. The solution is based on a microwave source emitting in a certain bandwidth. A continuous adjustment of this bandwidth allows a very fast adaptation of the incident signal to the changes in the resonance frequency of the measurement cell, which are a consequence of the variations in the sample. This way, materials undergoing large and abrupt changes in their dielectric properties during the heating process can be measured, and also the measurable temperatures range is increased above 1000 °C.

In [29], the authors describe the design of the reactor and the main devices of the system, together with the electromagnetic algorithms (cavity perturbation method) which were modified to obtain improved accuracy in the dielectric characterization. However, this equipment presents additional innovative features which allow performing these dielectric measurements in a way that was not possible before. The aim of this paper is precisely to explain these new features and show some of their practical implications with new experimental results. These additional features include the continuous control of sample volume during the process (for a precise determination of the sample dielectric properties), or the automatic adjustment of process parameters (power, bandwidth, heating rate) with a PID algorithm implemented in Labview.

To this end, the microwave system described is applied for the observation of thermal phenomena in different materials and high temperature processes, showing experimental results which are new and to the authors’ knowledge, are reported for the first time. The *in situ* dielectric characterization gives valuable information about the heating process as well as the peculiarities shown by the materials when microwave fields are applied under different conditions.

## 2. Microwave System Description

**Figure 1:**
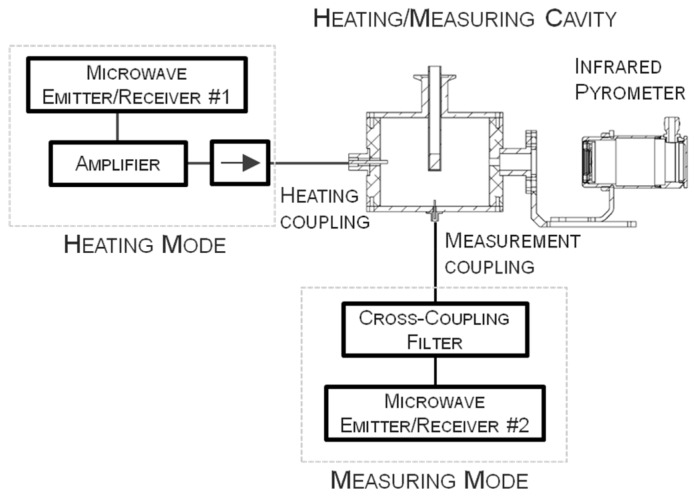
Schematic view of the equipment designed to determine microwave-matter interactions.

Figure 1 shows the schematic view of the microwave system designed for high temperature dielectric heating and permittivity measurements. The microwave cell is a cylindrical cavity designed to operate simultaneously in two modes, one for heating and the other one for measuring. The heating mode (TE_111_), designed to resonate near the ISM standard frequency of 2.45 GHz, is fed into the cavity by a probe inserted through the side wall, while the measuring mode (TM_010_) is fed by another probe through the bottom wall of the cavity. The source for the incident signal of the heating mode is a Vector Network Analyzer (VNA) connected to an amplifier. The VNA delivers an output of 0 dBm (1 mW) and the gain of the amplifier is around 50 dB. The maximum power delivered to the cavity in this configuration is 150 W. A second VNA is used as a source for the measuring mode, with a cross-coupling filter to avoid interferences between the heating and the measuring signals. The use of two VNAs can be a limiting factor because of their high cost. However, it is possible to use low-cost microwave transducers to act as VNAs in a limited frequency range [30]. In this case, the total cost of the system is notably reduced, as well as the volume occupied by the system.

The sample is placed in a quartz holder which is inserted in the cavity through a cylindrical waveguide. The cavity also has two holes in the side wall for sample inspection with a video camera and for temperature measurement with an external IR pyrometer. The dimensions of all the open accesses to the cavity are designed to avoid any leakage of microwave energy outside the cavity.

Since the pyrometer points to the surface of the quartz holder, a temperature calibration method has been previously performed to obtain the relationship between the bulk temperature of the sample and the pyrometer measurements [29].

Once tube and sample are positioned inside the cavity, a control software implemented in Labview allows setting the desired heating rate, monitoring the sample temperature, recording the video image of the sample and the response of the cavity, both for the measuring and heating modes. A PID algorithm adjusts the source bandwidth to follow the changes in the cavity response and obtain the desired heating rate throughout the process.

## 3. Results

The continuous measurement of the resonance in the cavity (resonant frequency and quality factor) gives the information for calculating the dielectric properties of the sample (the real part or dielectric constant and the imaginary part or loss factor) by cavity perturbation methods as a function of the temperature. With this procedure, the accuracy in dielectric properties determination has been estimated to be 3% for the dielectric constant and 10% for the loss factor.

**Figure 2:**
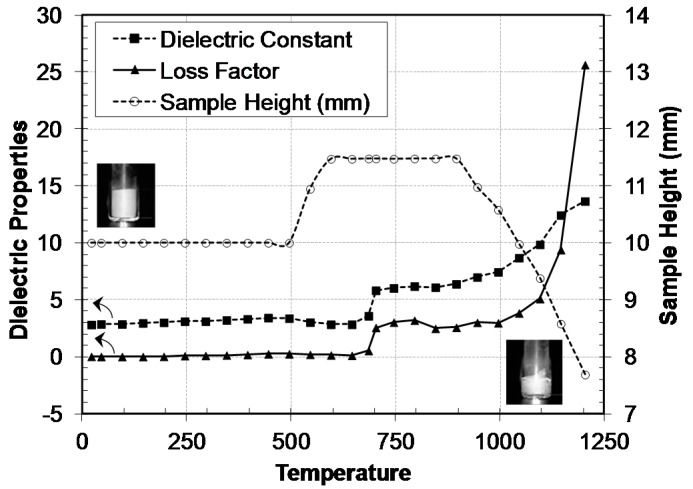
Dielectric properties of ceramic frit at microwave frequencies during the heating process up to 1200 °C.

An important source of error arises if the sample dimensions change during the process, and these changes are not properly accounted for. A deviation of 5% in the sample height can lead to errors up to 40% in the dielectric properties determination [29]. Thus, a precise control of the sample height is essential if changes are expected during the process. Figure 2 shows the dielectric properties of a ceramic frit sample together with the sample height up to 1200 °C. The sample height has been determined with an estimated precision of 0.1 mm from the analysis of video images recorded during the process (The inlets in Figure 2 represent two of these images: at room temperature and at 1200 °C).

The sample consists of a powder made with a mix of ceramic raw materials (mainly quartz, colemanite, dolomite, potassium feldspar and alumina) which are heated up to high temperatures (>1000 °C) in order to achieve a completely fluxing material.

The figure shows important variations in the sample height during the process, due to different reactions and transformations. At temperatures above 450 °C, the water inside the sample passes to a gas state. The gas tries to exit leading to the expansion of the sample. In contrast, a contraction takes place above 900 °C, due to the melting of the sample.

The dielectric properties of the sample slightly increase with increasing temperature up to 700 °C. At this temperature, the dolomite decomposition starts, and a sharp change is observed in dielectric constant and loss factor. A second increase begins about 900–1000 °C, due to the melting of the sample. The effect of the melting is more pronounced in the sample loss factor, reaching from very low values at room temperature to high values at the end of the process.

**Figure 3:**
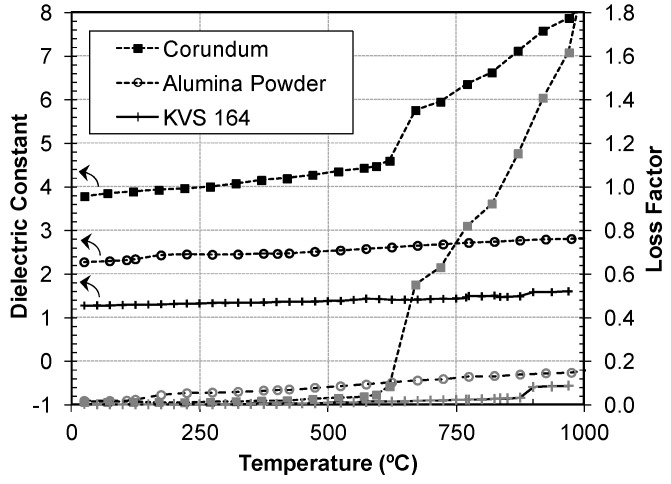
Dielectric properties of low loss materials at microwave frequencies during the heating process up to 1000 °C.

Figure 3 shows the dielectric properties of selected low loss materials. The first sample is corundum (loss factor of 0.0012 at 23 °C), the second sample is alumina powder (loss factor of 0.0016 at 23 °C). The third sample is KVS-164^®^, a ceramic material composed of a mix of alumina (65%) and silica (34%) fibers, used for high-temperature thermal insulation (loss factor of 0.0020 at 23 °C). Since these materials present a low loss factor at room temperature, they are almost transparent to microwave energy. They can be heated by microwave energy only if the cavity is carefully designed to concentrate the electromagnetic field in the sample volume.

The results show that the dielectric properties increase considerably with temperature. In the case of corundum, there is a sharp increase of several orders of magnitude in the loss factor at 600 °C, which converts corundum into a good microwave absorber from this temperature (loss factor of 2.1 at 1000 °C). The alumina sample has a smooth and gradual increase; its loss factor reaches 0.15 at 1000 °C. The material with the lowest loss factor within the entire temperature range is KVS-164. It presents a slight increase with temperature up to 900 °C, where the loss factor reaches the value 0.08.

Important conclusions can be extracted from this graph. For example, these three materials are good candidates to be used as transparent containers or insulators inside microwave applicators, at least at low temperatures. However, for high temperature applications, KVS-164 is the only one that presents low absorption of microwave energy at temperatures above 600–700 °C. The use of corundum could lead to an undesirable overheating or runaway effect due to its abrupt change at 600 °C. This dielectric characterization with temperature gives very useful information for microwave designers to select the best materials depending on the application.

**Figure 4:**
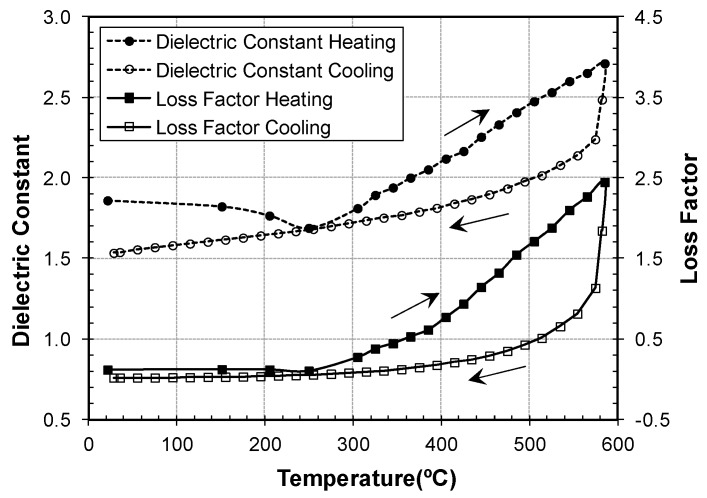
Dielectric properties of zeolite CBV100^®^ at microwave frequencies during the heating and cooling cycle.

Figure 4 illustrates the case of materials which present different dielectric properties for heating and cooling cycles. In this case, a sample of the commercial zeolite CBV100^®^ (type Y zeolite with silica/alumina mole ratio of 5.1) has been measured controlling the heating and cooling rates with the aid of the PID algorithm integrated in the software, which adjusts the microwave power applied to the sample. The maximum temperature was limited to 600 °C, to avoid any degradation of the zeolite.

The measurements of the dielectric constant during the heating cycle present a clear change of tendency at 250 °C, due to the loss of structural water contained in the zeolite. After that, the increase of both dielectric constant and loss factor presents a linear dependence with the temperature. However, the zeolite properties follow a different path when they are measured during the cooling cycle. This difference in the heating and cooling curves is maintained in subsequent cycles. The causes of this hysteresis are still under investigation, but show the importance of characterizing the materials during both heating and cooling cycles.

**Figure 5:**
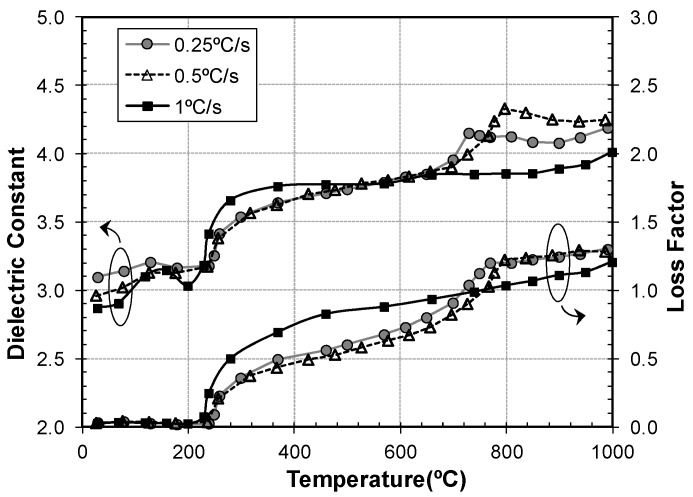
Dielectric properties of zirconia samples at microwave frequencies during the heating process at different heating rates (0.25 °C/s, 0.5 °C/s and 1 °C/s).

The possibility to control the heating and cooling rates with a specific rate allows the study of how these rates affect the kinetics of the thermal processes in materials. As an example, Figure 5 shows the dielectric properties of a Zirconia powder sample (TZ3YE^®^ partially stabilized with 3% mole of Yttria), heated at three different heating rates (0.25 °C/s, 0.5 °C/s and 1 °C/s).

The sample heated at 1 °C/s presents a more abrupt increase at 100 °C due to an accelerated loss of the moisture present in the sample. There is also a marked difference in the behavior of this sample around 226 °C, reaching higher dielectric constant and loss factors than the other two samples. In the cases where the heating rate is lower (0.5 °C/s and 0.25 °C/s), this increase in the dielectric properties occurs at slightly higher temperatures (236 °C).

In the situation of conventional heating, monoclinic zirconia converts to tetragonal at about 800°C, causing a further increase in the dielectric properties [31]. The results show that this process starts at lower temperatures in the case of microwave heating, being this observation in accordance with previous studies [32]. The figure also shows different onset temperatures depending on the heating rate. In the case of 0.25 °C/s and 0.5 °C/s, this transformation leads to abrupt changes at 728 °C and 796 °C respectively. However, the sample heated at 1 °C/s presents a gradual increase of its dielectric properties, matching the other curves at about 1000 °C, when the zirconia is supposed to be completely tetragonal.

These studies give valuable information about the kinetics of the thermal processes, helping to define the best heating conditions.

## 4. Discussion

Different materials and thermal processes induced by microwaves have been studied through the *in situ* characterization of their dielectric properties.

Materials with dielectric properties changing abruptly from very low to moderate and high have been accurately measured in a unique heating cycle. The precise control of the heating conditions makes it possible to quantify the effects of different parameters on the materials’ transformations and kinetics through the experiments. Unexpected hysteresis cycles were also encountered in some materials when studying heating and cooling cycles.

These characterizations were not possible before, especially when complex or mixtures of materials undergoing very abrupt changes at high temperatures were involved.

The results presented in this paper illustrate how this recent development allows predicting in a reliable way the behavior of materials under high frequency fields, which is essential to design and optimize new high temperature microwave processes.

Nevertheless, it is clear that a profound understanding on the underlying mechanisms needs further investigation. From the authors’ point of view, further steps comprise the *in situ* combination of other techniques for the microstructural analysis of the samples during the heating process, such as Raman spectroscopy. Correlations between dielectric properties and other physico-chemical characteristics will give more complete information about the different mechanisms affecting the microwave-matter interactions, especially at high temperatures.

## 5. Materials and Methods

Table 2 shows the different materials, suppliers and characteristics of the samples measured in this investigation.

For the measurements, each sample was placed in a quartz holder with inner diameter of 9.8 mm. To ensure an optimum material processing and characterization (uniform temperature distribution in the sample, maximum accuracy in dielectric properties determination, *etc.*), the height of the samples is limited to 15 mm. Since the level of compaction of powdered samples strongly affects the measurement of the dielectric properties, the weight was measured to calculate the apparent density of each sample (see Table 2).

In the case of KVS-164 board, a rod was machined with the same dimensions as the rest of samples (9.8 mm diameter and 15 mm height) and placed in the quartz holder.

The samples were measured as received except the zeolite which was previously dried at 100 °C during 5 h due to their hydrophilic nature (a high water content in the sample may mask the thermal processes during heating).

All the experiments were conducted in air atmosphere with the exception of the zeolite, which was treated in a Nitrogen atmosphere, to avoid any rehydration during the cooling cycles. 

Heating and cooling rates were set to ±10 °C/min except for the three zirconia samples, which were heated at 0.25 °C/s, 0.5 °C/s and 1 °C/s, respectively.

## Figures and Tables

**Table 1:** Summary of previous work done on dielectric characterization of materials with temperature.

Approach	References		Comments
Measurements at frequencies different from ISM standard frequencies (2.45 GHz, 915 MHz)	[5,6]		Measurements in the Hz, KHz, MHz range
	[7,8] around 3–4 GHz [9] from 8 to 12 GHz [10] from 8 to 40 GHz [11] from 7 to 18 GHz [12] at 6 GHz [13] from 7 to 13 GHz, [14] from 6 to 40 GHz [15] at 9 GHz		Measurements in the GHz range
Measurements at intermediate temperatures (< 800 °C)	[16] <100 °C [9] <500 °C	[17] <325 °C	Max. Temperature <500 °C
	[7,18] <600 °C	[10] <800 °C	Max. Temperature >500 °C
Measurements with conventional heating	[5,6,9,13,14,15,17,18,19,20,21,22,23,24,25]		Only the sample is heated
	[11]		The cavity and the sample are heated
Measurements with MW heating and mechanical tuning	[11,12,16,18,26,27]		One mode for heating and measurement
	[7,8,28]		Different modes for heating and measurement
Measurements with MW heating without mechanical tuning	[29]		–

**Table 2:** Characteristics of the samples measured in this work.

Material	Description	Supplier	Density (g/cm^3^)
Ceramic Frit WP187^®^	Raw materials mix (Powder)	Keraben, Barcelona, Spain	0.82
ALTRA^®^ KVS-164	Thermal insulation board (Solid) with Al_2_O_3_ (65%)-SiO2 (34%) fibers	Rath Inc., Newark, DE, USA	0.31
Corundum	Al_2_O_3_ (99.9%) (Solid)	CeramTech, Nuremberg, Germany	2.05
Calcined Alumina	Calcined at 1250 °C, soda content (3000 ppm of Na_2_O) (Powder)	Alteo, Gardanne, France	1.53
Zeolite CBV100^®^	Type Y zeolite with silica/alumina mole ratio of 5.1 (Powder)	Zeolyst CV, Farmsum, the Netherlands	0.51
Zirconia TZ3YE^®^	Partially stabilized with 3% mol of Yttria	Tosoh, Tokyo, Japan	1.42 (1 °C/s) 1.47 (0.5 °C/s) 1.48 (0.25 °C/s)

## Abbreviations

The following abbreviations are used in this manuscript:

IR: Infrared
ISM: Industrial Scientific and Medical
PDI: Proportional Derivative Integral
TE: Transversal Electric
TM: Transversal Magnetic
VNA: Vector Network Analyzer
